# Eating Disorders

**DOI:** 10.1186/1472-6874-4-S1-S21

**Published:** 2004-08-25

**Authors:** Enza Gucciardi, Nalan Celasun, Farah Ahmad, Donna E Stewart

**Affiliations:** 1 University Health Network Women's Health Program, University of Toronto, 657 University Avenue, Toronto, Canada; 2 University Health Network Women's Health Program, University of Toronto, 657 University Avenue, Toronto, Canada; 3 University Health Network Women's Health Program, University of Toronto, 657 University Avenue, Toronto, Canada; 4 University Health Network Women's Health Program, University of Toronto, 657 University Avenue, Toronto, Canada

## Abstract

**Health Issue:**

Eating disorders are an increasing public health problem among young women. Anorexia and bulimia may give rise to serious physical conditions such as hypothermia, hypotension, electrolyte imbalance, endocrine disorders, and kidney failure.

**Key Issues:**

Eating disorders are primarily a problem among women. In Ontario in 1995, over 90% of reported hospitalized cases of anorexia and bulimia were women. In addition to eating disorders, preoccupation with weight, body image and self-concept disturbances, are more prevalent among women than men.

Women with eating disorders are also at risk for long-term psychological and social problems, including depression, anxiety, substance abuse and suicide. For instance, in 2000, the prevalence of depression among women who were hospitalized with a diagnosis of anorexia (11.5%) or bulimia (15.4 %) was more than twice the rate of depression (5.7 %) among the general population of Canadian women. The highest incidence of depression was found in women aged 25 to 39 years for both anorexia and bulimia.

**Data Gaps and Recommendations:**

Hospitalization data are the most recent and accessible information available. However, this data captures only the more severe cases. It does not include the individuals with eating disorders who may visit clinics or family doctors, or use hospital outpatient services or no services at all. Currently, there is no process for collecting this information systematically across Canada; consequently, the number of cases obtained from hospitalization data is underestimated. Other limitations noted during the literature review include the overuse of clinical samples, lack of longitudinal data, appropriate comparison groups, large samples, and ethnic group analysis.

## Background

Eating disorders are an increasing public health problem among young women[1]: they may give rise to serious physical problems such as hypothermia, hypotension, electrolyte imbalance, endocrine disorders and kidney failure. Women with eating disorders are also at risk of long-term psychological and social problems, including depression, anxiety, substance abuse and suicide. The costs in terms of quality of life, loss of productivity, serious medical problems and mortality are high. [2]

Clinical eating disorders include anorexia nervosa and bulimia nervosa. [3] Anorexia is characterized by a severely calorie-restricted diet, resulting in a body weight that is at least 85% below that expected for age and height. [3] Bulimia is identified by frequent fluctuations in weight and recurrent episodes of compulsive bingeing followed by self-induced vomiting, purging, fasting, laxative use and/or excessive exercise in attempts to avoid weight gain.[3] Eating disorders not otherwise specified include behaviours such as chronic dieting, purging and binge-eating, which do not meet the full criteria for a specific eating disorder,[3] they are two to five times as common as the clinical eating disorders.

Eating disorders are, by and large, a problem among women. From the data collected in the Ontario Health Survey, Mental Health Supplement, the lifetime prevalence of bulimia (according to the criteria of the Diagnostic and Statistical Manual, 3rd revision [DSM-III-R]) among women aged 15 to 65 was estimated as 1.1% in 1990.[4] In 1995, 95% of reported hospitalized cases of anorexia and more than 90% of hospitalized cases of bulimia in Ontario were women.[5]

In addition to eating disorders, preoccupation with weight and body image, and self-concept disturbances, are more prevalent among women than men.[4,6-9] Personal, behavioural and socio-environmental factors, such as negative body image, low self-esteem, fear of becoming fat, chronic dieting and social pressures to be thin, are identified risk factors.[1,6,10-13]

### Body Image

Body image concerns and preoccupation with body weight and shape increase as girls become older and more aware of the idealized societal preference for a thin body shape.[14] The images of women in the media and popular culture place pressure on vulnerable young girls and women to live up to these expectations, regardless of their natural body shape.[8,9] In British Columbia, it was found that by age 18, 80% of girls at all weights reported that they would like to weigh less.[15,16] A school-based population study involving 1,739 adolescent women aged 12 to 18 years in Toronto, Hamilton and Ottawa found that current dieting to lose weight was reported by 23% of participants, binge-eating by 15%, self-induced vomiting by 8.2% and the use of diet pills by 2.4%.[17]

Body shape dissatisfaction and preoccupation with weight are not limited to adolescents but also occur in children. A recent Canadian school-based study concluded that 34% of prepubescent girls, 36% of early pubescent girls and 76% of post-pubescent girls were dissatisfied with their body shape.[11] In a survey of eating and smoking behaviours among boys and girls in grades 4 to 8 in southwestern Ontario and Charlottetown, more than 25% from each grade reported not eating breakfast every day, and there was a sharp increase among girls beginning in grade 7.[18] Unhealthy eating patterns in childhood can adversely affect health, contribute to chronic disease in later life, and often persist into adolescence and adulthood, since change is difficult once eating patterns are established.[18]

### Morbidity

The starvation associated with anorexia and the chronic vomiting frequently associated with bulimia can cause serious medical problems, such as hypothermia, hypotension, anemia, osteoporosis, endocrine abnormalities, dehydration, kidney stones, metabolic alkalosis and dental caries.[1] Girls and women with eating disorders are also at increased risk of menstrual irregularities such as amenorrhea, infertility, [19,20] miscarriages and fetal complications such as prematurity, low birth weight, malformations and low Apgar scores.[1,20-23]

Mothers who have or have had an eating disorder may also create abnormal behavioural patterns when feeding their children, such as irregular feeding schedules, detached non-interactive mealtimes, and use of food for non-nutritive purposes, which may lead to second-generation eating problems. [24-27]

### Psychological Morbidity

In addition to depression, anxiety and obsessive-compulsive disorders, eating disorders are also associated with diminished libido, altered sleeping patterns, irritability and suicide attempts.[3,28,29] Ontario women with bulimia have higher levels of anxiety, depression and alcohol abuse than those without bulimia.[3,4,29,30] Smoking and substance abuse are much more prominent among teenaged girls with eating disorders than among those with healthy eating habits. [3,17,28] In a recent analysis of the 1997 Ontario Student Drug Use Survey, adolescent females who perceived themselves as overweight were almost 50% more likely to smoke than those who considered themselves of average weight or too thin, whereas weight perceptions were not associated with smoking among males.[31] Several studies have suggested an association between a traumatic experience (sexual or physical abuse) and later self-injury. A recent study found that among patients with eating disorders there is a more than 30% lifetime risk of self-injurious behaviour.[32]

### Mortality

The rate of death from anorexia is higher than from bulimia because of the complications of starvation and electrolyte imbalances, or suicide.[33,34] A recent review reported a mortality rate of 0.6% for anorexia as compared with 0.3% for bulimia.[35] A longitudinal U.S. study (21-year follow-up) of 84 women with anorexia reported that 14 women (16.7%) had died, and 12 of the 14 had died of causes directly related to anorexia; the observed death rate was 9.8 times greater than expected.[6]

### Socio-Economic Status

Although previous studies in Canada and the United States have demonstrated differences in education and socio-economic status (SES) in the prevalence of obesity, [36-38] the relation between eating disorders and SES is still unclear.[17,39] Jones et al[17] observed that SES was not significantly associated with disturbed eating behaviours in a school-based Ontario population (n = 530), findings that are consistent with those of previous studies and may reflect the pervasive influence of the media on all SES groups.[39]

### Ethnic Subgroups

Cultural beliefs and attitudes are identified as significant contributing factors in the development of eating disorders.[40] Canadian research on eating disorders and ethnic background, however, is extremely limited.

A few studies propose that cultural beliefs may actually protect ethnic groups against eating disorders, but their effect may be eroded as adolescents face pressures of acculturation.[41] A recent study of Mexican-American women across generations reported that second-generation women displayed the most disordered eating patterns and the highest degree of acculturation to mainstream U.S. culture.[42] Experiences of cultural change (such as those of immigrants, for example) may also increase vulnerability to eating disorders.[43,44]

### This Study

This study presents data from the NPHS and national databases to investigate the burden of eating disorders in Canada and to explore the differences in attitude to weight between men and women.

## Methods

### NPHS

Within the NPHS, 1996–1997 (see Appendix A for details about the methods of data collection for the NPHS), the population selected for this analysis consisted of women and men aged 20 to 64, excluding pregnant women and people less than 3 feet (0.9 metres) or greater than 6 feet 11 inches (2.1 metres) in height. All data were weighted to represent the total population at the date of each survey.

Respondents who perceived themselves to be either underweight or overweight were asked to state their desired weight. The responses were analyzed according to four categories of body mass index (BMI) – underweight (BMI < 20), acceptable weight (BMI 20–25), some excess weight (BMI 25–27) and overweight (BMI > 27).

### CIHI

From the CIHI database, any separations from hospital with an ICD-9 code of 307.1 for anorexia and 307.5 for bulimia and other unspecified disorders of eating from 1994 to 1999 were extracted. Crude hospitalization rates, age-specific rates, and age-standardized hospitalization rates per 100,000 separations across provinces and territories were examined. Furthermore, age-specific rates for the comorbidity of depressive disorder (ICD-9 code of 311) among those with a hospital separation for an eating disorder were also examined for the year 2000.

## Results

### Prevalence

The prevalence of eating disorders is difficult to ascertain, since many people may not even be aware that they have an eating disorder, are reluctant to seek medical care or have not been hospitalized. Although epidemiologic research can approximate the prevalence of eating disorders within a population, the lack of standard methodologies and community samples results in conflicting estimates.

Hospitalization data are the most recent and accessible information available, but only the more severe cases are captured and not the individuals with eating disorders who may visit clinics or family doctors, use hospital outpatient services or use no services at all. Currently, there is no process for collecting this information systematically across Canada; consequently, the number of cases obtained from hospitalization data is an underestimate.

**Figure 1:**
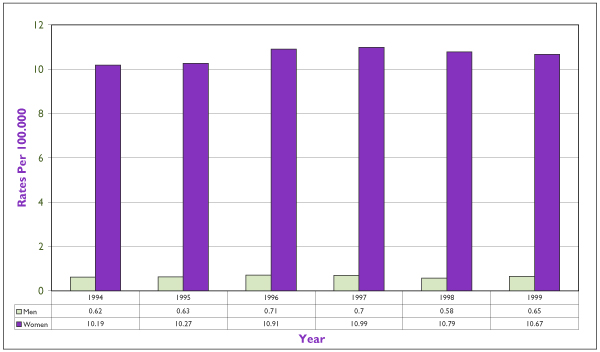
**Canadian Inpatient Crude Hospital Separation Rates per 100,000 for Anorexia, Bulimia and Eating Disorder Not Otherwise Specified, 1994–1999 **Source: Canadian Institute for Health Information

Inpatient crude hospital separations in Canada for any diagnosis of anorexia, bulimia and other unspecified eating disorders appear to have increased somewhat (by 4.7%), from 10.2 to 10.7 per 100,000 women between 1994 and 1999 (Figure 1). Although the rates among men were notably lower, they also increased slightly (4.8%) during the same period, from 0.6 to 0.7 per 100,000 men (Figure 1). These increases may be due to better awareness and detection of eating disorders and improved specialized inpatient treatment programs.

**Figure 2:**
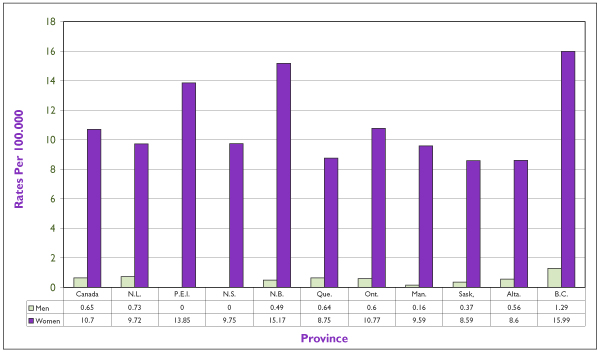
**Inpatient Crude Hospital Separation Rates per 100,000 for Anorexia, Bulimia and Eating Disorder Not Otherwise Specified by Canadian Province, 1994–1999 **Source: Canadian Institute for Health Information

Across Canada, age-standardized hospital separation rates for eating disorders were highest among women in British Columbia (15.9 per 100,000 women) and New Brunswick (15.1), and lowest in Saskatchewan (8.6) and Alberta (8.6) (Figure 2).

### Differences between Men and Women

**Figure 3:**
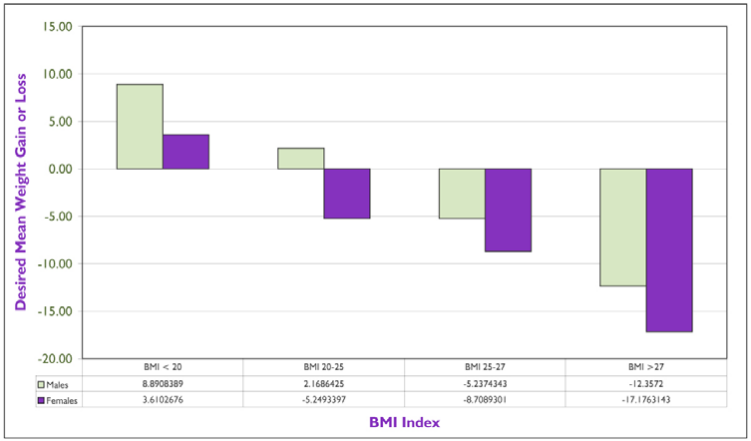
**Desired Mean Weight Gain or Loss by Body Mass Index Category and Sex **Source: Statistics Canada, National Population Health Survey 1996–1997

The 1996–1997 NPHS data suggest that more women than men wished to weigh much less than their actual weight (see Figure 3). Among those who had an acceptable BMI, between 20 and 25, women wanted to lose an average of 5.3 kg, whereas men wanted to gain an average of 2.2 kg. Among those with a BMI between 25 and 27 or greater than 27, women wished to achieve a higher average weight loss than men (8.7 kg versus 5.2 kg and 17.2 kg versus 12.4 kg, respectively). Similarly, among those who were underweight, with a BMI of less than 20, the average desired weight gain was smaller for women than for men (3.6 versus 8.9 kg).

### Depression

**Figure 4:**
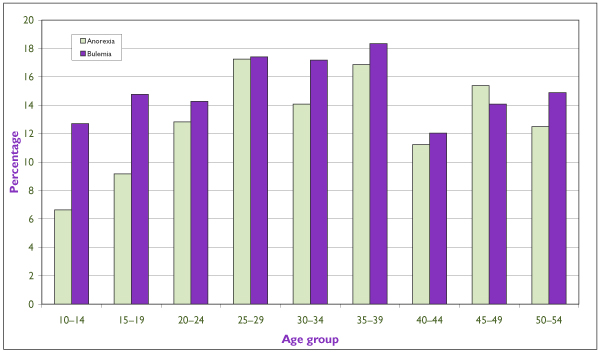
**Canadian Inpatient Age-specific Prevalence for Depression Among Women with Anorexia, Bulimia and Eating Disorder Not Otherwise Specified, 2000 **Source: Canadian Institute for Health Information

In 2000, the prevalence of depression among women who were hospitalized with a diagnosis of anorexia (11.54%) or bulimia (15.36%) was more than twice the prevalence of depression (5.7%) in the general population of women, according to the 1998–1999 NPHS.[45] (For depression rates of the general population please see the chapter on "Depression".) The highest prevalence of depression was found among women between 25 and 39 years of age for both anorexia and bulimia (see Figure 4).

### Vulnerable Subgroups

**Figure 5:**
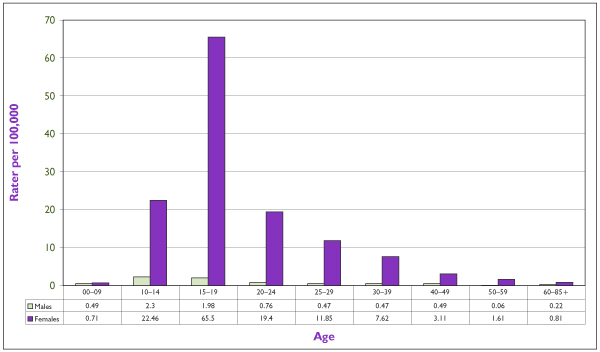
**Canadian Inpatient Age-Specific Hospital Separation Rates per 100,000 for Anorexia, Bulimia and Eating Disorders Not Otherwise Specified, 1994–1999 **Source: Canadian Institute for Health Information

Although distorted body images and eating disorders are experienced by women of all ages, the highest rates of hospitalization for eating disorders occur among young women. Age-specific hospital separation rates are highest among adolescent women aged 15 to 19 years (65.5 per 100,000 women) followed by those aged 10 to 14 years. The rates slowly decrease with age (Figure 5). Many young women do not meet the criteria for an eating disorder, but they may still show sub-clinical symptoms (disordered eating behaviours) that can harm their health. [7-9]

## Discussion

### Data Limitations

Several limitations were noted during the literature review: lack of longitudinal data, of appropriate comparison groups, of large samples and of ethnic group analysis. Most studies were based on clinical samples, making it difficult to generalize findings to the general population. Furthermore, many of these studies were cross-sectional and limited in their ability to make causal links.

CIHI hospital separation data provide information on the number of times each diagnosis is made and not on the number of people with the diagnosis, which leads to duplicate reporting. Comparable international rates of eating disorders were unobtainable. Nevertheless, two recommendations can be made.

## Recommendations

• Data should be collected on eating disorders in Canadian women, including the prevalence and risk factors in subgroups such as disabled, immigrant and visible minority women.

• Cross-cultural and longitudinal evaluations of attitudes and behaviours towards disordered eating should be conducted in large community samples to monitor trends, examine protective and non-protective factors, and assist in the development and planning of preventive and treatment programs.
